# Supplementary material for: Stenotrophomonas maltophilia exhibits defensive multicellularity in response to a Pseudomonas aeruginosa quorum sensing molecule
Source: bioRxiv. 2025 May 2:2025.05.02.651457. Preprint. [Version 1] doi: 10.1101/2025.05.02.651457 (PMC12190822; doi:10.1101/2025.05.02.651457)
Supplement: Supplement 1 [file media-1.pdf]

SUPPLEMENTARY FIGURES

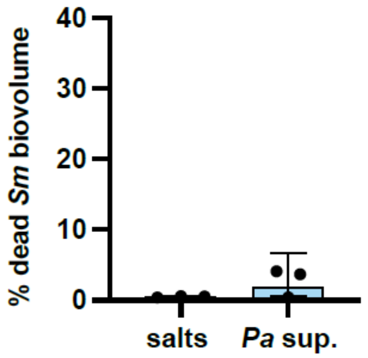

Supp. Figure 1. *Pa* secreted products do not cause significant *Sm* death. *Sm* cells were exposed to 50% (v/v) media salts or *Pa* cell-free supernatant for 2 h in static 96-well plates and the biovolume of PI-stained (dead) bacteria was quantified as a percentage of the total biovolume of GFP-tagged bacteria. Data shown are the mean ± SD for three biological replicates. Significance was tested for comparison to the salts control by an unpaired *t*-test (difference was not significant).

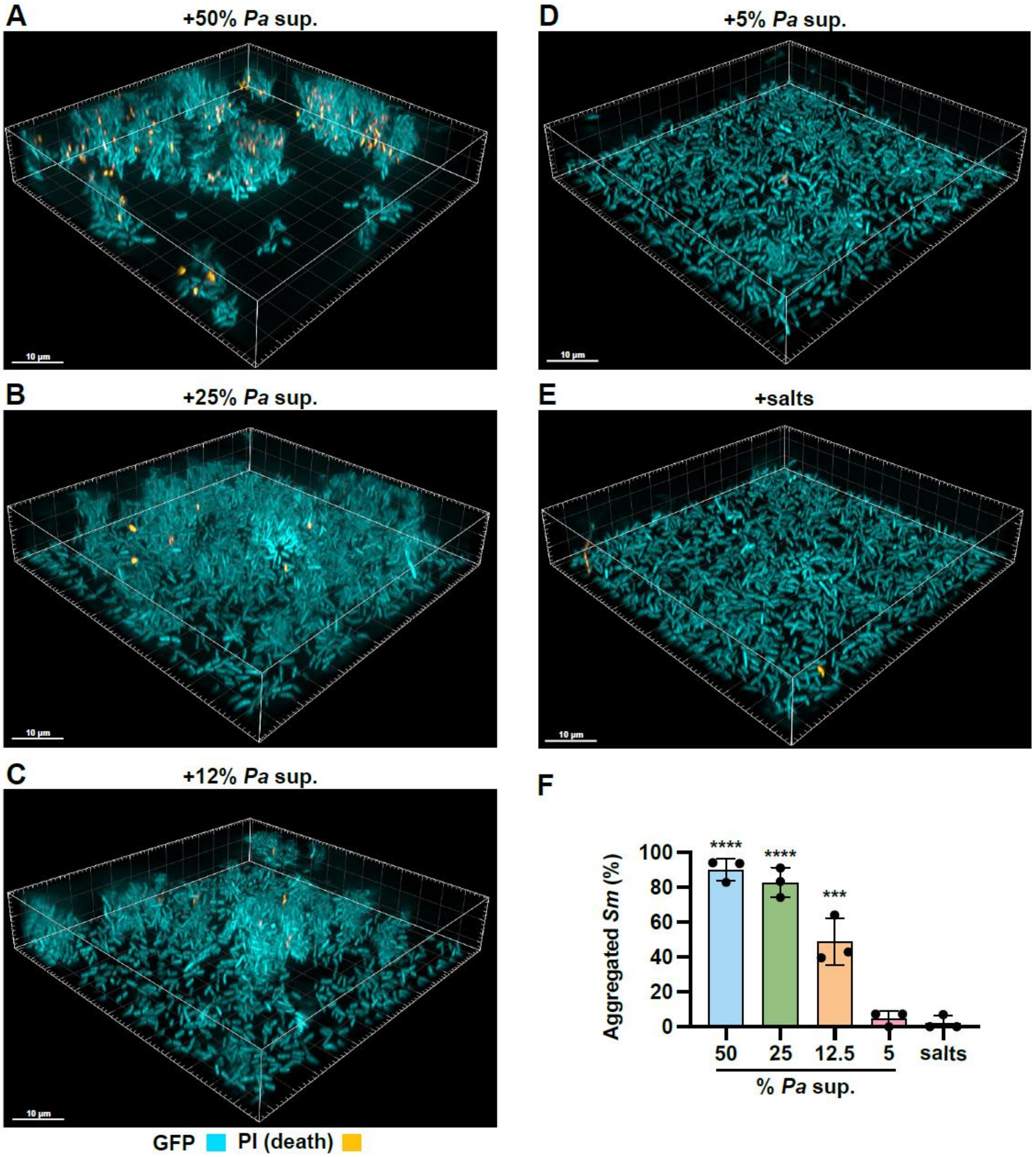

Supp. Figure 2. *Pa* supernatant-induced *Sm* aggregation is a dose-dependent response. (A-E) Fluorescent microscopy of GFP tagged and PI-stained *Sm* in static 96-well plates exposed for 2 h to 50, 25, 12.5, or 5% *Pa* cell-free supernatant or salts control, respectively. Representative images of three biological replicates are shown. **(F)** *Sm*

aggregate biovolume derived from representative images of **A-E** and quantified as a percentage of the total *Sm* biovolume. Data shown are the mean ± SD for three biological replicates. Significance is shown for comparison to the salts control, as tested by a one-way ANOVA, followed by the Tukey's test for multiple comparisons (***, $p < 0.001$; ****, $p < 0.0001$).

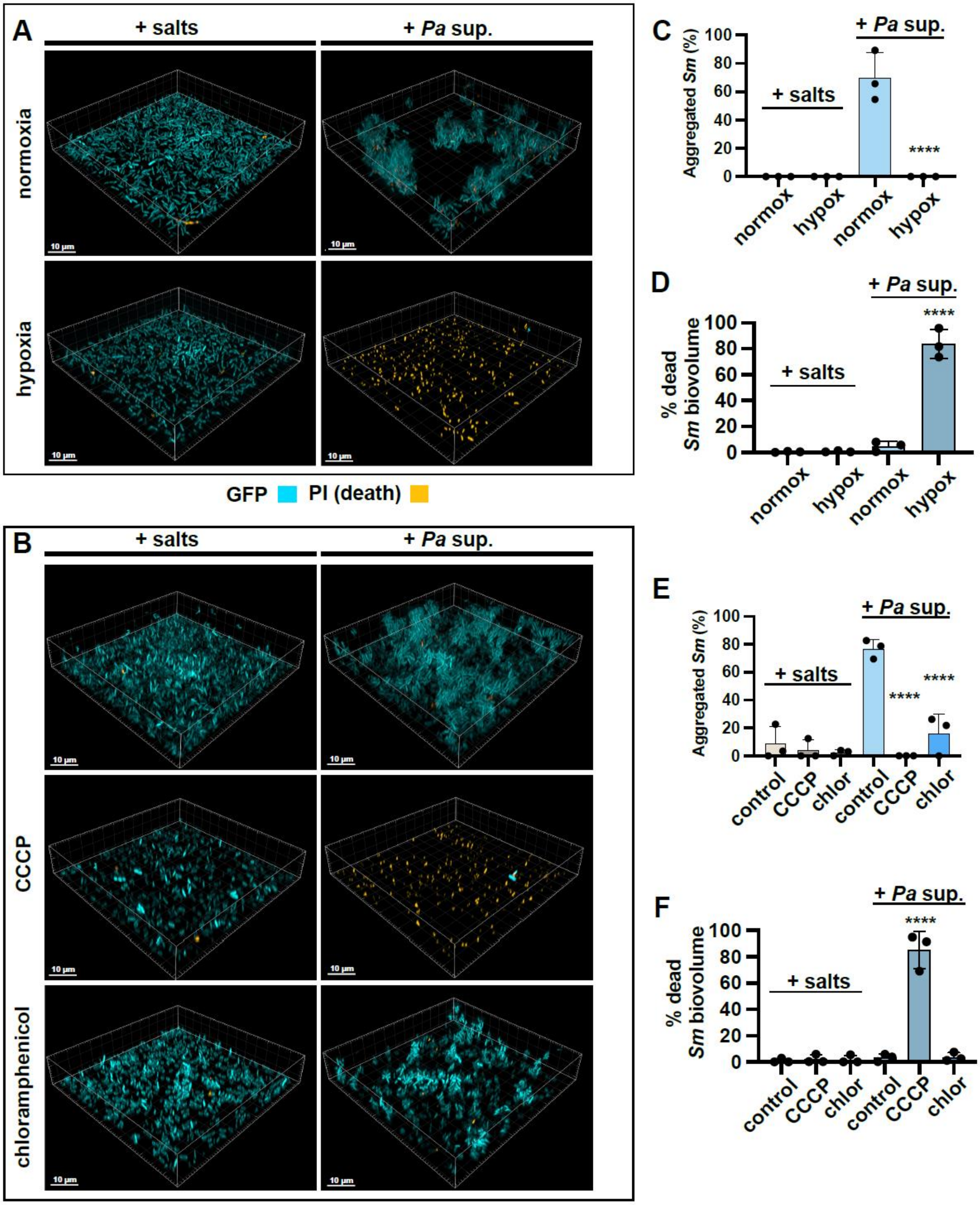

Supp. Figure 3. *Sm* aggregation in response to *Pa* supernatant is an active behavior. GFP-tagged *Sm* was stained with PI and pre-treated for 20 minutes with **(A)**

hypoxia and **(B)** CCCP or chloramphenicol and subsequently exposed to *Pa* cell-free supernatant for 2 h and imaged in static 96-well plates. Representative images of three biological replicates are shown. **(C, E)** Biovolume of the resulting *Sm* aggregates was measured as a percentage of the total *Sm* biovolume. **(D, F)** Biovolume of dead *Sm* cells was measured as a percentage of the total *Sm* biovolume. **(C-F)** Data shown are the mean ± SD for three biological replicates. Significance is shown for comparison to **(C, D)** the respective normoxia condition, or **(E, F)** the untreated *Pa* supernatant control, as tested by a one-way ANOVA with the Tukey's test for multiple comparisons (****, $p < 0.0001$).

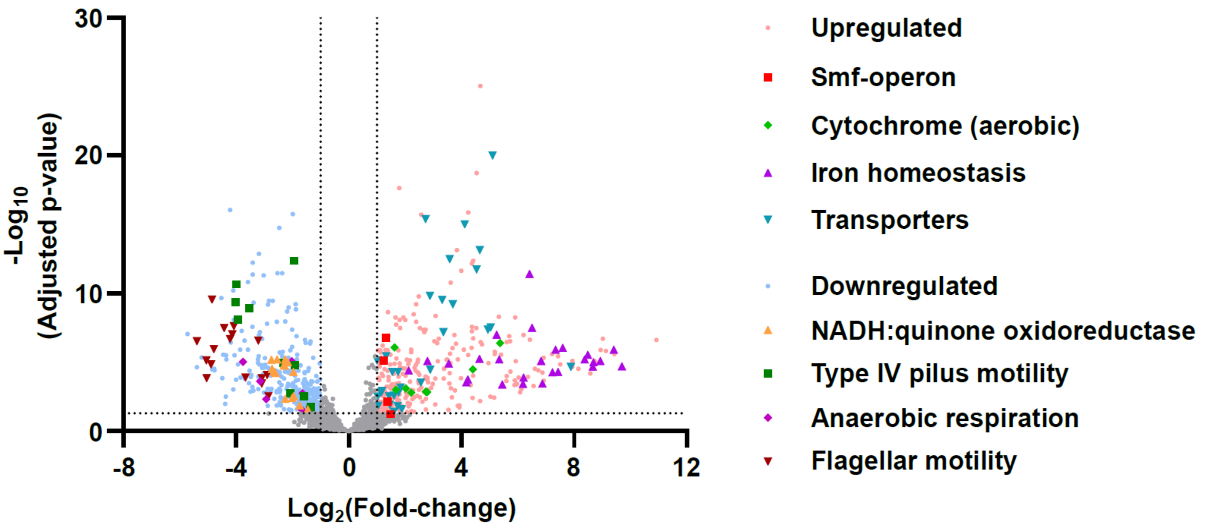

Supp. Figure 4. *Sm* upregulates the *smf-1* operon and differentially regulates respiration and motility pathways upon exposure to *Pa* supernatant. WT *Sm* cells were exposed for 30 minutes to either the salts control or *Pa* cell-free supernatant, and transcript levels were measured. Shown are the $\log_{10}$(adjusted *p*-values) and $\log_2$(fold-change).

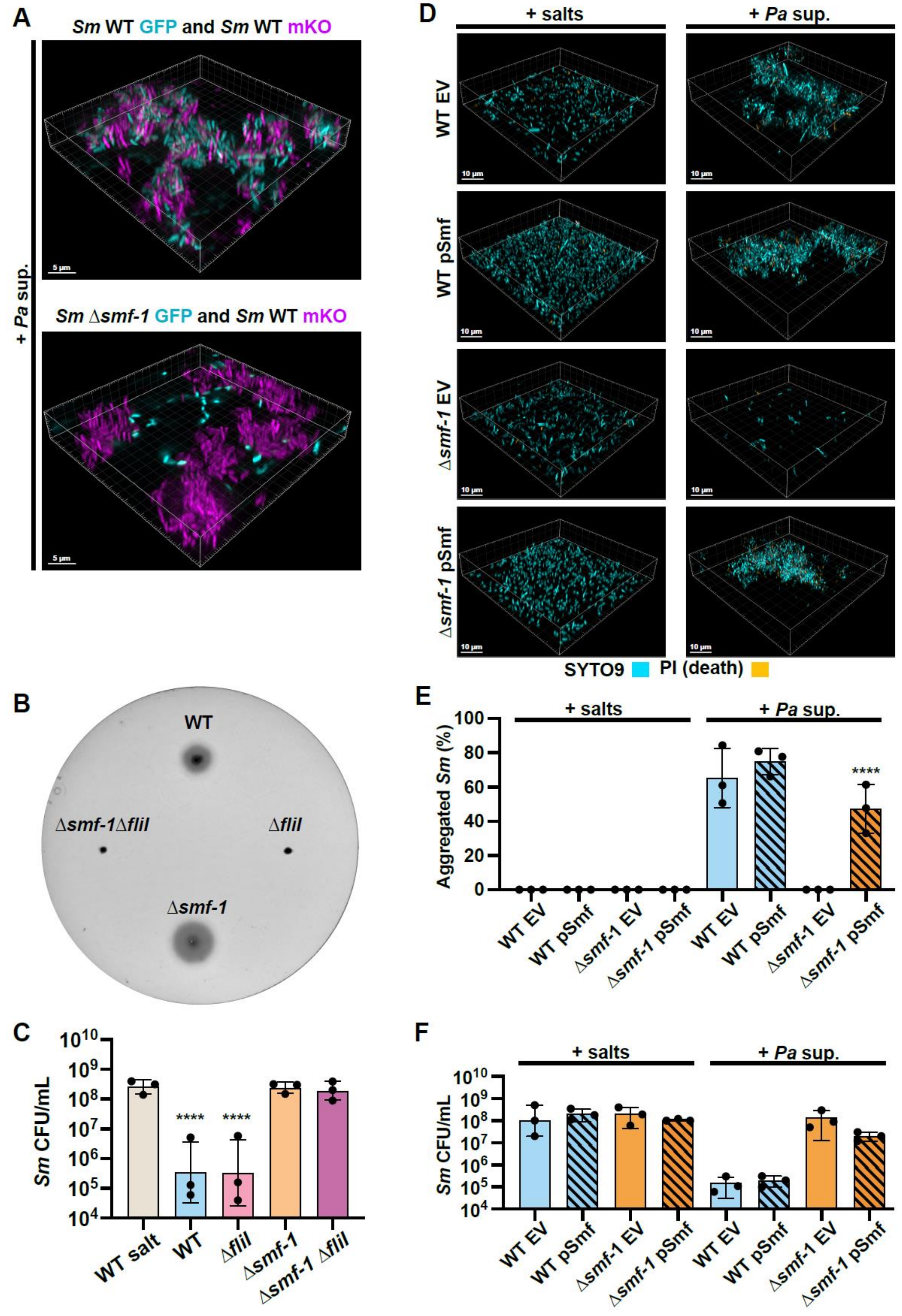
A
Sm WT GFP and Sm WT mKO
+ Pa sup.
5 µm
Sm Δsmf-1 GFP and Sm WT mKO
5 µm
B
WT
Δsmf-1Δflil
Δflil
Δsmf-1
C
Sm CFU/mL
10^10
10^9
10^8
10^7
10^6
10^5
10^4
****
****
WT salt
WT
Δflil
Δsmf-1
Δsmf-1 Δflil
D
+ salts
+ Pa sup.
WT EV
WT pSmf
Δsmf-1 EV
Δsmf-1 pSmf
10 µm
SYTO9
PI (death)
E
+ salts
+ Pa sup.
Aggregated Sm (%)
100
80
60
40
20
0
****
WT EV
WT pSmf
Δsmf-1 EV
Δsmf-1 pSmf
WT EV
WT pSmf
Δsmf-1 EV
Δsmf-1 pSmf
F
+ salts
+ Pa sup.
Sm CFU/mL
10^10
10^9
10^8
10^7
10^6
10^5
10^4
WT EV
WT pSmf
Δsmf-1 EV
Δsmf-1 pSmf
WT EV
WT pSmf
Δsmf-1 EV
Δsmf-1 pSmf

Supp. Figure 5. *Sm* aggregation requires the Smf-1 fimbriae and is motility-independent. (A) Microscopy of 1:1 mixtures of WT mKO with either WT GFP or Δ*smf-1* GFP in 96-well plates after 2 h exposure to *Pa* cell-free supernatant. **(B)** Swim motility of WT, Δ*fliI*, Δ*smf-1* and Δ*smf-1* Δ*fliI* on soft agar plates imaged after 24 h. **(C)** CFU enumeration of WT, Δ*fliI*, Δ*smf-1* and Δ*smf-1* Δ*fliI* after 2 h exposure to salts control and *Pa* supernatant. **(D-F)** WT and Δ*smf-1* containing either the empty vector (EV) or one containing the *smf-1* operon (pSmf) after 2 h of exposure to salts and *Pa* supernatant were stained with SYTO9 and PI and assessed via **(D)** microscopy and **(E)** biovolume quantification, or **(F)** CFU enumeration. **(A, B, D)** Representative images of three biological replicates are shown. **(C, E, F)** Data shown are the mean ± SD for three biological replicates. Significance is shown for comparison to **(C)** the WT salts control, or **(E, F)** the EV control, as tested by a one-way ANOVA with the Tukey's test for multiple comparisons (***, $p < 0.001$; ****, $p < 0.0001$).

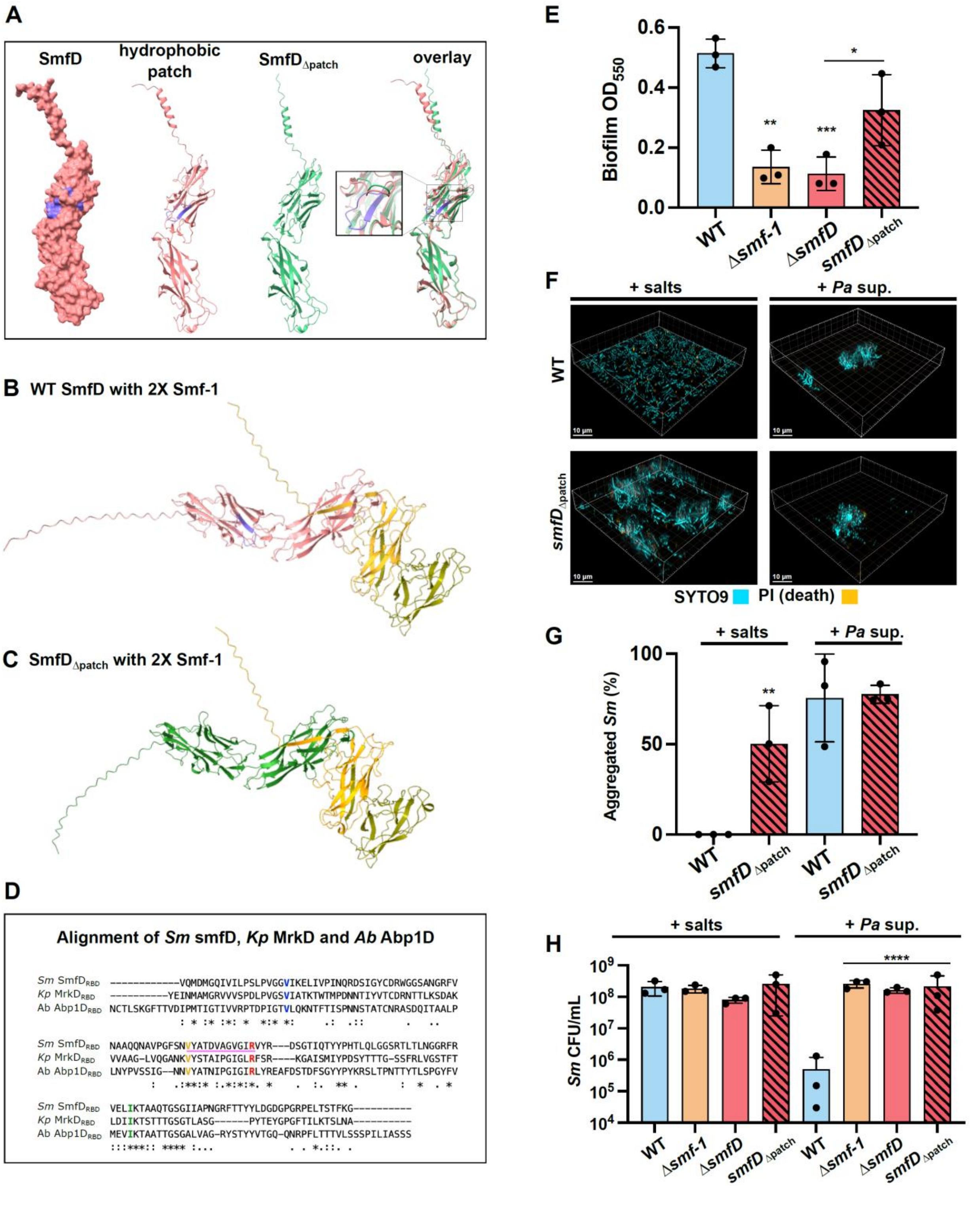

Supp. Figure 6. SmfD hydrophobic patch alters *Sm* aggregation. (A) (L to R): SmfD space-filling model, SmfD ribbon drawing with hydrophobic patch depicted in purple,

SmfD hydrophobic patch deletion mutant, and the overlay, with the zoomed-in inset showing the deleted stretch. **(B, C)** Predicted complex model of two Smf-1 subunits, colored in yellow and olive, and **(B)** single WT SmfD or **(C)** single SmfD$_{\Delta \text{patch}}$. **(D)** Alignment of receptor binding domains (RBD) of *S. maltophilia* SmfD, *K. pneumoniae* MrkD and *A. baumannii* Abp1D. Residues shown to be required for collagen binding in *K. pneumoniae* MrkD are depicted in blue, green, yellow and red. Region deleted in the *smfD*$_{\Delta \text{patch}}$ mutant is underlined in purple and contains two conserved residues from the putative hydrophobic patch. **(E)** Quantification of WT, Δ*smf-1*, Δ*smfD,* and *smfD*$_{\Delta \text{patch}}$ biofilm production. **(F, G)** WT, and *smfD*$_{\Delta \text{patch}}$ cells were exposed to the salts control and *Pa* supernatant for 2 h in static 96-well plates, stained with SYTO9 and PI, and analyzed via **(F)** microscopy and **(G)** aggregate biovolume. **(H)** CFU enumeration of WT, Δ*smf-1*, Δ*smfD,* and *smfD*$_{\Delta \text{patch}}$ cells exposed to the salts control and *Pa* cell-free supernatant for 2 h. **(F)** Representative images of three biological replicates are shown. **(E, G, H)** Data shown are the mean ± SD for three biological replicates. Significance is shown for comparison to the respective WT condition, or the indicated comparison, as tested by a one-way ANOVA with the Tukey's test for multiple comparisons (*, $p < 0.05$; **, $p < 0.01$; ***, $p < 0.001$).

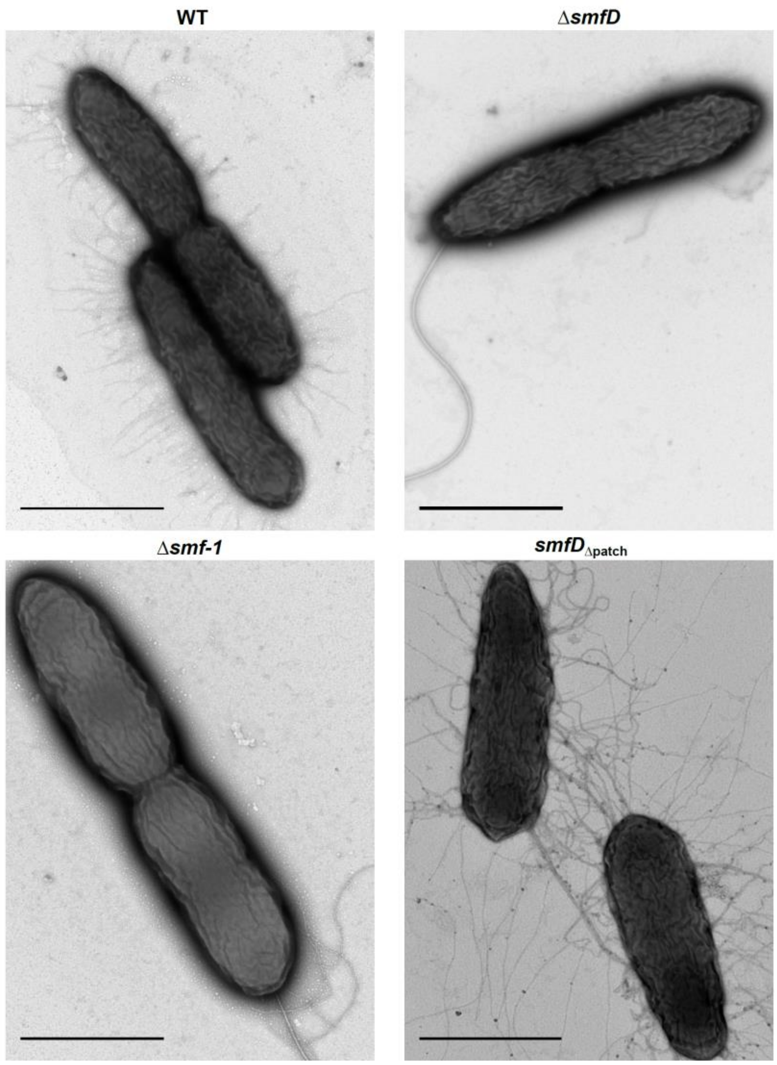

Supp. Figure 7. The Δ*smf-1* and Δ*smfD* mutants lack fimbriae, while the *smfD*$_{\Delta\text{patch}}$ mutant shows longer, tangled fimbrial structures. Negative-stained transmission electron micrographs of early log WT, Δ*smf-1*, Δ*smfD,* and *smfD*$_{\Delta\text{patch}}$ mutant cells. Scale bars; 1 µm.

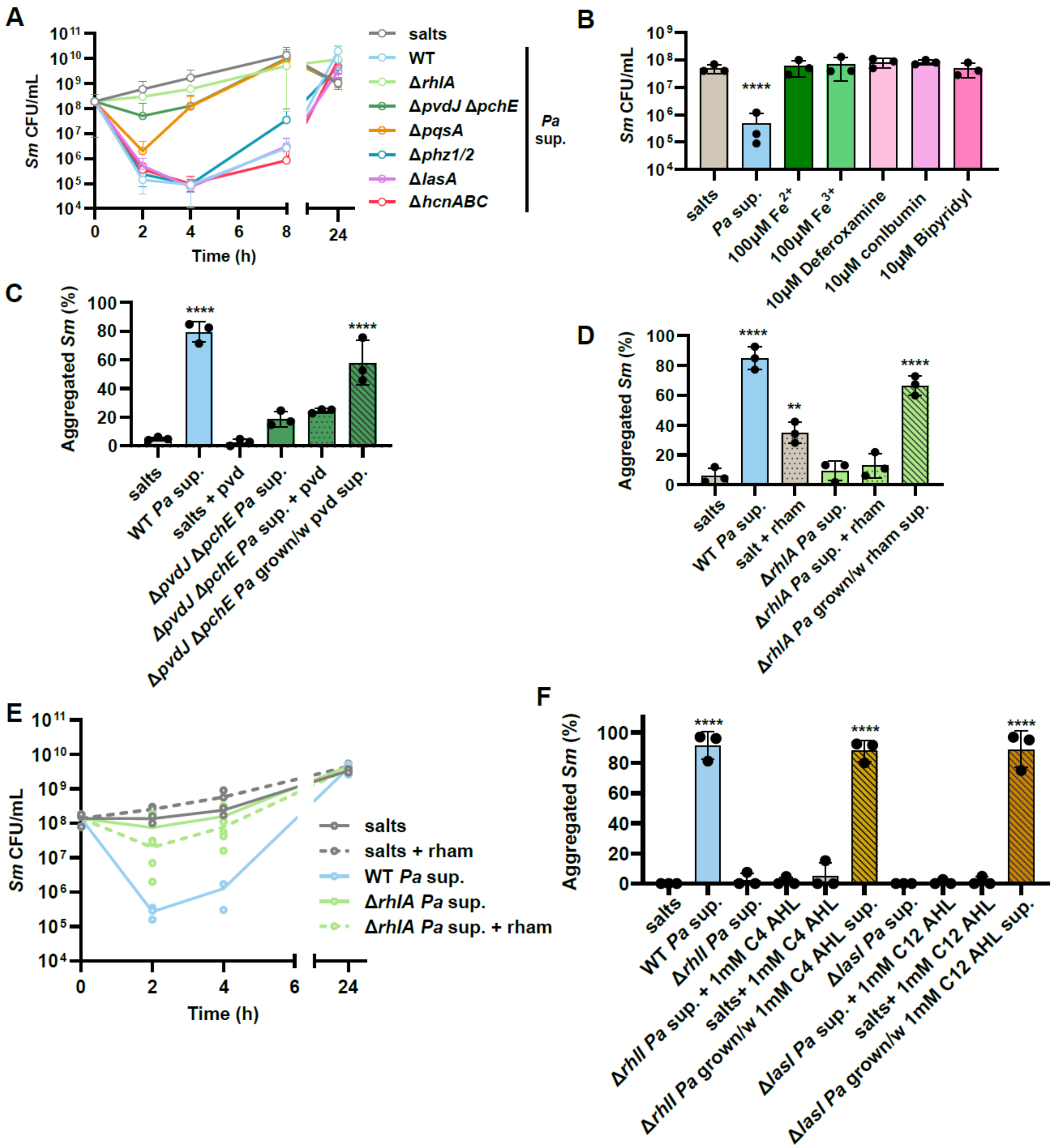

Supp. Figure 8. *Pa* siderophores, rhamnolipids and N-acyl-homoserine lactone quorum sensing molecules indirectly mediate *Sm* aggregation. (A) CFU enumeration of *Sm* aggregation in response to cell-free supernatant derived from *Pa* WT and mutants deficient for the production of rhamnolipids (Δ*rhlA*), siderophores (Δ*pvdJ*

Δ*pchE*), alkylquinolones (Δ*pqsA*), phenazines (Δ*phz1/2*), the LasA protease (Δ*lasA*) and hydrogen cyanide (Δ*hcnABC*)**. (B)** *Sm* aggregation as measured by CFU enumeration after 2 h exposure to salts, WT *Pa* supernatant, exogenous supplementation of ferrous or ferric iron, or the chelators ferrozine, conalbumin and 2,2 -Bipyridyl**. (C)** Biovolume quantification of *Sm* aggregation in static 96-well plates in response to salts with or without exogenous pyoverdine (pvd), *Pa* WT supernatant, Δ*pvdJ* Δ*pchE* supernatant with or without supplemental pyoverdine, and supernatant from the Δ*pvdJ* Δp*chE* mutant grown with pyoverdine supplementation. **(D)** Biovolume quantification of *Sm* aggregation after 2 h exposure to salts with or without exogenous rhamnolipids (rham), *Pa* WT supernatant, Δ*rhlA* supernatant with or without supplemental rhamnolipids, and supernatant from Δ*rhlA* grown with exogenous rhamnolipids**. (E)** CFU enumeration at the indicated time-points of *Sm* exposed to salts with or without exogenous rhamnolipids, WT *Pa* supernatant, and Δ*rhlA* supernatant with or without exogenous rhamnolipids. **(F)** Biovolume quantification of *Sm* aggregation in response to salts with or without N-butanoyl-L-homoserine lactone (C4 AHL) or N-dodecanoyl-L-homoserine lactone (C12 AHL), *Pa* WT supernatant, Δ*rhlI* supernatant with or without exogenous C4 AHL, supernatant from Δ*rhlI* grown with exogenous C4 AHL, Δ*lasI* supernatant with or without exogenous C12 AHL, and supernatant from Δ*lasI* grown with exogenous C12 AHL. Data shown are the mean ± SD for three biological replicates. Significance is shown for comparison to **(B-D, F)** the salts control, as tested by a one-way ANOVA with the Tukey's test for multiple comparisons (****, $p < 0.0001$). Data for **(B)** was log-transformed prior to statistical testing.

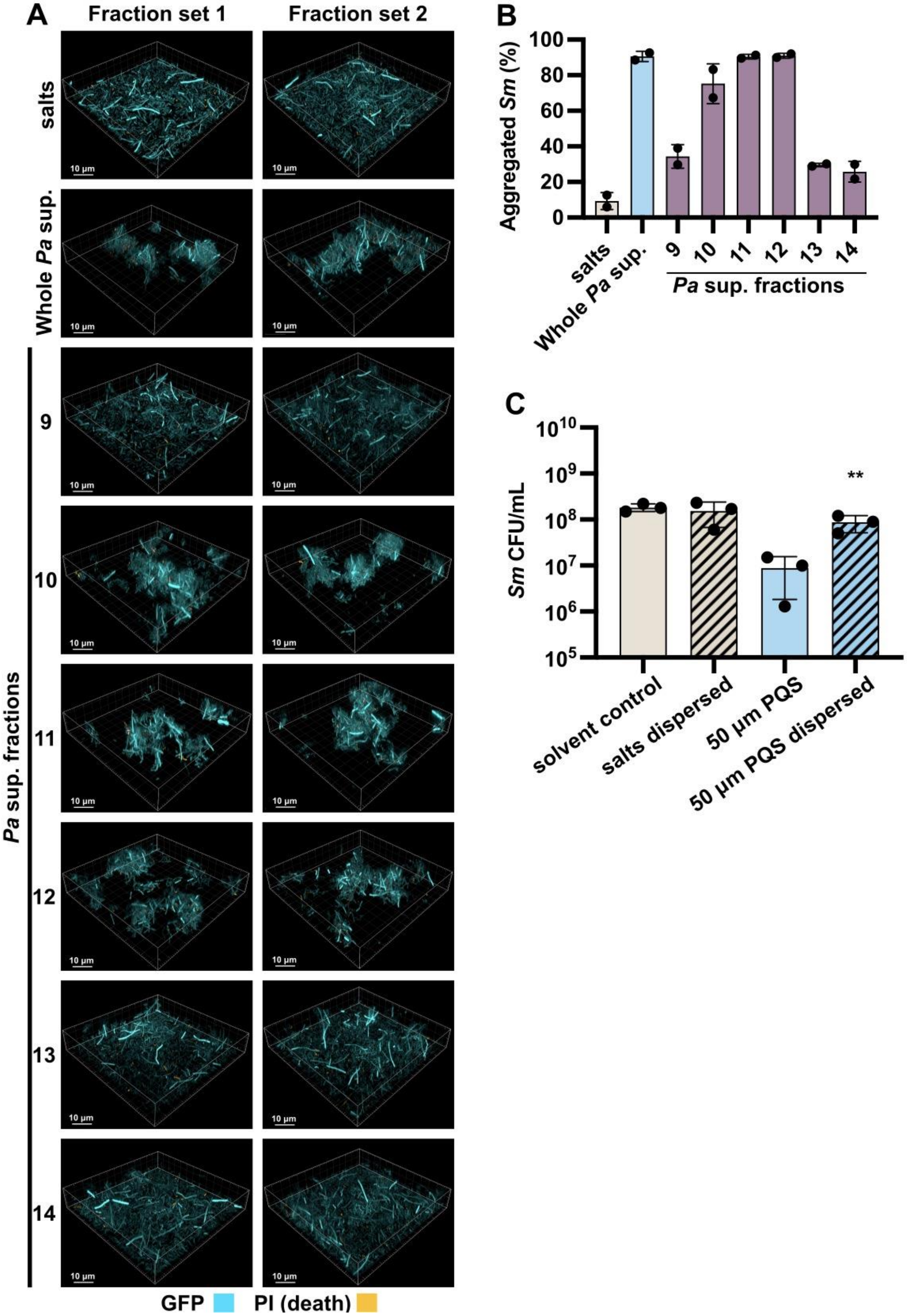
A
Fraction set 1
Fraction set 2
salts
Whole Pa sup.
Pa sup. fractions
9
10
11
12
13
14
10 µm
GFP
PI (death)
B
Aggregated Sm (%)
100
80
60
40
20
0
salts
Whole Pa sup.
9
10
11
12
13
14
Pa sup. fractions
C
Sm CFU/mL
10^{10}
10^9
10^8
10^7
10^6
10^5
**
solvent control
salts dispersed
50 µm PQS
50 µm PQS dispersed

Supp. Figure 9. Specific *Pa* supernatant fractions and PQS induce *Sm* aggregation. **(A)** Fluorescent microscopy of GFP tagged and PI-stained *Sm* exposed for 2 h in static 96-well plates to M63 salts medium control, whole *Pa* supernatant, and *Pa* supernatant fractions 9 through 14. Two independent biological replicates (fraction sets) are shown **(B)** Biovolume of the resulting *Sm* aggregates was measured as a percentage of the total *Sm* biovolume. **(C)** CFU enumeration of *Sm* after 2 h of exposure to salts or salts supplemented with 50 μM exogenous PQS followed by mechanical dispersion. Data shown are the mean ± SD for **(B)** two fractions sets and **(C)** three biological replicates. **(C)** Significance is shown for comparison to the respective non-dispersed control, as tested, after log transformation, by a one-way ANOVA with the Tukey's test for multiple comparisons (**, $p < 0.01$).

SUPPLEMENTARY TABLES

Supplementary Table 1. Mutations in evolved *S. maltophilia* isolates.

Strains[a]	ORF	Description	Annotation	Position	Mutation
**Population 1**					
E1.5.1	SMLT_RS03255 ← | → *purL*	Chitinase/ Phosphoribosylformylglycinamidine synthase	Intergenic (-324/-489)	704,573	C → T
	SMLT_RS03350 ← | → *smf-1*	Hypothetical protein/ Fimbrial protein	Intergenic (-324 / -136)	734,943	T → G
E1.5.2	*smf-1*	Fimbrial protein	coding (49/540 nt)	735,127	(CGCTCCGC)$_{1\to2}$
E1.5.3	SMLT_RS03350 ← | → *smf-1*	Hypothetical protein/ Fimbrial protein	Intergenic (-324 / -136)	734,943	T → G
E1.10.1	*smf-1*	Fimbrial protein	Coding (293/540 nt)	735,371	Δ1 bp
	SMLT_RS10875	Chemotaxis protein CheW	E119E	2,305,421	C → T
E1.10.2	SMLT_RS03350 ← | → *smf-1*	Hypothetical protein/ Fimbrial protein	Intergenic (-324 / -136)	734,943	T → G
	SMLT_RS14115 ← | ← SMLT_14120	Hypothetical protein/ Hypothetical protein	Intergenic (-343/+377; -345/+375)	3,013,208; 3,013,210	C → A; C → T
E1.10.3	SMLT_RS03255 ← | → *purL*	Chitinase/ Phosphoribosylformylglycinamidine synthase	Intergenic (-315/-498)	704,564	A → G
	SMLT_RS03350 ← | → *smf-1*	Hypothetical protein/ Fimbrial protein	Intergenic (-324 / -136)	734,943	T → G
	hda → | ← SMLT_RS05480	DNA regulatory inactivator/ Nucleotidyltransferase family protein	Intergenic (+366/+133)	1,174,552	G → T
	purB → | → SMLT_RS15205	Adenylosuccinate lyase/ cupin domain-containing protein	Intergenic (+416/-240)	3,235,912	C → G
**Population 2**					
E2.5.1	SMLT_RS01300 → | ← SMLT_RS01305	Sensor histidine kinase/ Zinc-dependent peptidase	Intergenic (+381/+220; +552/+49)	295,776; 295,947	C → A; C → A
	smf-1	Fimbrial protein	coding (344-349/540 nt)	735,422	(AGCTGC)$_{2\to1}$

	SMLT_RS09025	Phage portal protein	V449A	1,917,865	A → G
E2.5.2	SMLT_RS03350 ← | → *smf-1*	Hypothetical protein/ Fimbrial protein	Intergenic (-324 / -136)	734,943	T → G
	SMLT_RS09025	Phage portal protein	V449A	1,917,865	A → G
E2.5.3	SMLT_RS03350 ← | → *smf-1*	Hypothetical protein/ Fimbrial protein	Intergenic (-324 / -136)	734,943	T → G
	sufD	Fe-S cluster assembly protein	G115A	1,198,365	C → G
	SMLT_RS09025	Phage portal protein	V449A	1,917,865	A → G

[a]First position numeral indicates population 1 or 2; second numeral indicates isolation on 5th or 10th day of passaging; third numeral indicates isolate designation.

Supp. Table 4. Bacterial strains and plasmids used in this study

Strain	Description	Source
S. maltophilia		
SB148	K279a	(*1*)
SB490	K279a GFP	This study
SB629	K279a mkO	This study
SB292	K279a *smf-1*Δ_{1bp}	This study
SB586	K279a Δ*smf-1*	This study
SB630	K279a Δ*smf-1* GFP	This study
SB589	K279a Δ*smfD*	This study
SB587	K279a *smfD*$_{\Delta patch}$	This study
SB500	K279a Δ*fliI*	This study
SB501	K279a Δ*smf-1* Δ*fliI*	This study
SB609	K279a pBBR1MCS EV	This study
SB611	K279a pBBR1MCS pSmf	This study
SB610	K279a Δ*smf-1* pBBR1MCS EV	This study
SB615	K279a Δ*smf-1* pBBR1MCS pSmf	This study
AMT0482-08	*Sm* CF clinical isolate CF077	CFF Isolate Core
AMT0492-03	*Sm* CF clinical isolate CF082	CFF Isolate Core
AMT0492-08	*Sm* CF clinical isolate CF087	CFF Isolate Core
AMT0492-12	*Sm* CF clinical isolate CF091	CFF Isolate Core
P. aeruginosa		
1A3	PA14	(*2*)
SB489	PA14 mKO	(*3*)
SB92	PA14 Δ*rhlA*	(*3*)
SB459	PA14 Δ*rhlI*	(*4*)
AK625	PA14 Δ*pvdJ* Δ*pchE*	(*5*)
AK619	PA14 Δ*pqsA*	(*5*)
AK652	PA14 Δ*pqsH*	This study
AK660	PA14 Δ*pqsR*	This study
SB521	PA14 Δ*pqsR* mKO	This study
AK681	PA14 Δ*phz1/2*	(*5*)
SB97	PA14 Δ*lasA*	This study
SB460	PA14 Δ*lasI*	(*4*)
SB96	PA14 Δ*hcnABC*	This study
AMT0150-17	*Pa* CF clinical isolate CF002	CFF Isolate Core
AMT0457-07	*Pa* CF clinical isolate CF033	CFF Isolate Core
AMT0458-02	*Pa* CF clinical isolate CF047	CFF Isolate Core
AMT0492-02	*Pa* CF clinical isolate CF081	CFF Isolate Core
E. coli		
DH5α	*E. coli* strain used for cloning	NEB
S17-1 λ-pir	*E. coli* strain used for conjugation	(*6*)
AK193	*E. coli* UTI clinical isolate UTI-H	(*7*)
AK194	*E. coli* UTI clinical isolate UTI-P	(*7*)
Other species		

JE2	USA300_FPR3757 (CA-MRSA)-JE2	(*8*)
SB81	*Salmonella enterica*	ATCC 29630
SB480	*Burkholderia cenocepacia*, K56-2	J. Goldberg
SB575	*Achromobacter xylosoxidans*, CF clinical isolate	D. Limoli
SB576	*Achromobacter xylosoxidans*, CF clinical isolate	D. Limoli
SB577	*Achromobacter xylosoxidans*, ATCC 27061	D. Limoli
**Plasmids**		
pDONRP EX18Gm	Shuttle vector with attP sites and ccdB; Cm^r $Gent^r$	(*9*)
pEX18ApGW	Gateway-compatible gene replacement vector; Amp^R Cm^R	(*10*)
PCR8/GW/TOPO	Gateway entry vector; $Spec^R$	Invitrogen
pFLP2	FLP recombinase expressing plasmid; Amp^R $Carb^R$	(*11*)
pSB321	pDONRPEX18Gm: Δ*smf-1*; $Gent^r$	This study
pSB495	pDONRPEX18Gm: Δ*smfD*; $Gent^r$	This study
pSB630	pDONRPEX18Gm: $smfD_{\Delta patch}$; $Gent^r$	This study
pSB499	pDONRPEX18Gm: Δ*fliI*; $Gent^r$	This study
pSB601	pBBR1MCS: empty vector, Cm^r	(*12*)
pSB614	pBBR1MCS: pSmf operon complement, Cm^r	This study
pAK646	pEX18ApGW-Δ*pqsH*; $Gent^r$	This study
pAK647	pEX18ApGW-Δ*pqsR*; $Gent^r$	This study
pAK959	pEX18ApGW-Δ*hcn*; $Gent^r$	This study
pAK960	pEX18ApGW-Δ*lasA*; $Gent^r$	This study

Supp. Table 5. Primers used in this study

Description	Sequence 5′→3′
smf-1 Δ1bp F	GGGGACAAGTTTGTACAAAAAAGCAGGCTCAAAACACGTCGGCTTACAGGT
smf-1 Δ1bp R	GGGGACCACTTTGTACAAGAAAGCTGGGTAGTATCGGCGGTCTGGTTG
smf-1 Δ1bp. sequencing F	CTGCGCCAGGTCTTCGAG
smf-1 Δ1bp. sequencing R	CCGGAGAAGATCAGTCGCAG
Δ*smf-1*, upstream F	GGGGACAAGTTTGTACAAAAAAGCAGGCTCAAAACACGTCGGCTTACAGGT
Δ*smf-1*, upstream R	GGGTACGGCTACGATCAGTTCTTGTGCATTCGCTTTTACC
Δ*smf-1*, downstream F	GGTAAAAGCGAATGCACAAGAACTGATCGTAGCCGTACCC
Δ*smf-1*, downstream R	GGGGACCACTTTGTACAAGAAAGCTGGGTAGTCGTTGATGGTGATGAAGC
Δ*smf-1*, sequencing F	CTGCGCCAGGTCTTCGAG
Δ*smf-1*, sequencing R	GAGGCGGATGGTGTGTTC
Δ*smfD*, upstream F	GGGGACAAGTTTGTACAAAAAAGCAGGCTCACAATACCGTCACCCTCGACC
Δ*smfD*, upstream R	CTGGGGCCGCCTTACTCGATCATCGGCATGCTGCCT
Δ*smfD*, downstream F	AGGCAGCATGCCGATGATCGAGTAAGGCGGCCCCAG
Δ*smfD*, downstream R	GGGGACCACTTTGTACAAGAAAGCTGGGGTTCATTTCCTCCGGCCAC
Δ*smfD*, sequencing F	AGCGTGGTCGTGCATCC
Δ*smfD*, sequencing R	GACCTGCGTGTAGAGCGC
smfDΔ_{patch}, upstream F	GGGGACAAGTTTGTACAAAAAAGCAGGCTCACGCTTCGATACCCGCAAG
smfDΔ_{patch}, upstream R	GGTGCCCGAATCACGATAGACGTTGCTGAAACCCGGGAC
smfDΔ_{patch}, downstream F	GTCCCGGGTTTCAGCAACGTCTATCGTGATTCGGGCACC
smfDΔ_{patch}, downstream R	GGGGACAAGTTTGTACAAAAAAGCAGGCTCAGCGGATGCCGATCTTGCT
smfDΔ_{patch}, sequencing F	GGTGCCCGAGCCCTTGAA
smfDΔ_{patch}, sequencing R	GGTATTGCCGTGGCCAGC
Δ*fliI*, upstream F	GGGGACAAGTTTGTACAAAAAAGCAGGCTCAGCAGATCGAAGGCATCCTCG
Δ*fliI*, upstream R	GGCTTAACTCTCTTGTTCCACCAGGTTCATGCATTGGCTCCGGT
Δ*fliI*, downstream F	ACCGGAGCCAATGCATGAACCTGGTGGAACAAGAGAGTTAAGCC
Δ*fliI*, downstream R	GGGGACCACTTTGTACAAGAAAGCTGGGCGCACCGATATCGTCCAT
Δ*fliI*, sequencing F	GACTGCATCCGGATGACATC
Δ*fliI*, sequencing R	CAACCGGTCGAGGAAAGC
pSmf complementation insert F	GCCCGGGGACACGTTCTCTGGTTCGGTG
pSmf complementation insert R	TAGTGGATCCTTACTCGTACTGGATGGTGAAGGTGG

pSmf complementation vector F	GTACGAGTAAGGATCCACTAGTTCTAGAGCGGC
pSmf complementation vector R	AACGTGTCCCCGGGCTGCAGGA
Δ*pqsH*, A_F	GCTCATCGGTTACCTCTTGCAC
Δ*pqsH*, A_R_Gent	TCAGAGCGCTTTTGAAGCTAATTCGAAGAACGGTCATCCGTT GCTC
Δ*pqsH*, B_F_Gent	AGGAACTTCAAGATCCCCAATTCGGAGATGGCCGCACAGTA GC
Δ*pqsH*, B_R	ACGGTGACTACCACGACCTTG
Δ*pqsR*, A_F	CGGATTCTAACCGCATAGGTC
Δ*pqsR*, A_R_Gent	TCAGAGCGCTTTTGAAGCTAATTCGAATAGGCATCCCTTATT CCTTTTATTG
Δ*pqsR*, B_F_Gent	AGGAACTTCAAGATCCCCAATTCGGCCGCACCAGAGTAGAG C
Δ*pqsR*, B_R	CGAGGAAACCCGCAACAAGG
Δ*lasA*_A_F	GAGGTGGAAGCCGAGTTTTC
Δ*lasA*_A_R_Gm	TCAGAGCGCTTTTGAAGCTAATTCGCATGGGTAGCTCCTG GTC
Δ*lasA*_B_F_Gm	AGGAACTTCAAGATCCCCAATTCGTTGTACAACCCCGGC CTG
Δ*lasA*_B_R	ACCACCGGCATCATC TTC
Δ*hcn*_A_F	CGA GCT TTTCCCCTTCACC
Δ*hcn*_A_R_Gm	TCAGAGCGCTTTTGAAGCTAATTCGAAGGTGCATTGCCCT TTC
Δ*hcn*_B_F_Gm	AGGAACTTCAAGATCCCCAATTCGTGCTAGGTCCGCGAG GGGTAAATC
Δ*hcn*_B_R	CGAGCCACAACTGGTACAGC
GentR_F	CGAATTAGCTTCAAAAGCGCTCTGA
GentR_R	CGAATTGGGGATCTTGAAGTTCCT

REFERENCES

1. M. B. Avison, C. S. Higgins, C. J. Von Heldreich, P. M. Bennett, T. R. Walsh, Plasmid location and molecular heterogeneity of the L1 and L2 β-lactamase genes of *Stenotrophomonas maltophilia*. *Antimicrob Agents Chemother* **45**, 413 (2001).

2. L. G. Rahme, E. J. Stevens, S. F. Wolfort, J. Shao, R. G. Tompkins, F. M. Ausubel, Common virulence factors for bacterial pathogenicity in plants and animals. *Science (1979)* **268**, 1899–1902 (1995).

3. D. L. Warrell, T. M. Zarrella, C. Machalek, A. Khare, Interspecies surfactants serve as public goods enabling surface motility in *Pseudomonas aeruginosa*. *J Bacteriol* **206** (2024).

4. D. A. Hogan, Å. Vik, R. Kolter, A *Pseudomonas aeruginosa* quorum-sensing molecule influences *Candida albicans* morphology. *Mol Microbiol* **54**, 1212–1223 (2004).

5. A. Khare, S. Tavazoie, Multifactorial competition and resistance in a two-species bacterial system. *PLoS Genet* **11**, e1005715 (2015).

6. V. de Lorenzo, K. N. Timmis, Analysis and construction of stable phenotypes in gram-negative bacteria with Tn5- and Tn10-derived minitransposons. *Methods Enzymol* **235**, 386–405 (1994).

7. N. Cerca, T. Maira-Litrán, K. K. Jefferson, M. Grout, D. A. Goldmann, G. B. Pier, Protection against *Escherichia coli* infection by antibody to the *Staphylococcus aureus* poly-N-acetylglucosamine surface polysaccharide. *Proc Natl Acad Sci U S A* **104**, 7528–7533 (2007).

8. A. D. Kennedy, M. Otto, K. R. Braughton, A. R. Whitney, L. Chen, B. Mathema, J. R. Mediavilla, K. A. Byrne, L. D. Parkins, F. C. Tenover, B. N. Kreiswirth, J. M. Musser, F. R. DeLeo, Epidemic community-associated methicillin-resistant *Staphylococcus aureus*: recent clonal expansion and diversification. *Proc Natl Acad Sci U S A* **105**, 1327–1332 (2008).

9. L. R. Hmelo, B. R. Borlee, H. Almblad, M. E. Love, T. E. Randall, B. S. Tseng, C. Lin, Y. Irie, K. M. Storek, J. J. Yang, R. J. Siehnel, P. L. Howell, P. K. Singh, T. Tolker-Nielsen, M. R. Parsek, H. P. Schweizer, J. J. Harrison, Precision-engineering the *Pseudomonas aeruginosa* genome with two-step allelic exchange. *Nat Protoc* **10**, 1820–1841 (2015).

10. K. H. Choi, H. P. Schweizer, An improved method for rapid generation of unmarked *Pseudomonas aeruginosa* deletion mutants. *BMC Microbiol* **5** (2005).

11. T. T. Hoang, R. R. Karkhoff-Schweizer, A. J. Kutchma, H. P. Schweizer, A broad-host-range F1p-FRT recombination system for site-specific excision of chromosomally-located DNA sequences: Application for isolation of unmarked *Pseudomonas aeruginosa* mutants. *Gene* **212**, 77–86 (1998).

12. M. E. Kovach, P. H. Elzer, D. Steven Hill, G. T. Robertson, M. A. Farris, R. M. Roop, K. M. Peterson, Four new derivatives of the broad-host-range cloning vector pBBR1MCS, carrying different antibiotic-resistance cassettes. *Gene* **166**, 175–176 (1995).
